# Predominant Mechanisms in the Treatment of Wastewater Due to Interaction of Benzaldehyde and Iron Slag Byproduct

**DOI:** 10.3390/ijerph17010226

**Published:** 2019-12-28

**Authors:** Ayad A. H. Faisal, Saif S. Alquzweeni, Laith A. Naji, Mu Naushad

**Affiliations:** 1 Department of Environmental Engineering, College of Engineering, University of Baghdad, Baghdad-10011, Iraq; add.ali.lith@gmail.com; 2 Department of Civil Engineering, College of Engineering, University of Babylon, Babylon-51001, Iraq; saifalquzweeni@gmail.com; 3 Department of Chemistry, College of Science, Bld#5, King Saud University, Riyadh-11451, Saudi Arabia

**Keywords:** benzaldehyde, iron slag, adsorption mechanism, cation bridge

## Abstract

Iron slag is a byproduct generated in huge quantities from recycled remnants of iron and steel factories; therefore, the possibility of using this waste in the removal of benzaldehyde from contaminated water offers an excellent topic in sustainability field. Results reveal that the removal efficiency was equal to 85% for the interaction of slag and water contaminated with benzaldehyde at the best operational conditions of 0.3 g/100 mL, 6, 180 min, and 250 rpm for the sorbent dosage, initial pH, agitation time, and speed, respectively with 300 mg/L initial concentration. The maximum uptake capacity of iron slag was 118.25 mg/g which was calculated by the Langmuir model. Physical sorption may be the major mechanism for the removal of benzaldehyde onto iron slag based on the analysis of isotherm and kinetic sorption data and thermodynamically, the process was spontaneous and endothermic. Finally, the X-ray fluorescence spectroscopy (XRF), X-ray diffraction (XRD), Fourier transform infrared (FT-IR), scanning electron microscope (SEM) and energy-dispersive spectroscope (EDS) tests for reactive material certified that the dissolution of calcium oxide can enhance the removal of benzaldehyde by the formation of bridge cations.

## 1. Introduction

Benzaldehyde is one of the most industrially useful members of the aromatic aldehydes family. It may exist in combined form such as apricot, glycoside in almond, peach seeds and cherry as well as being utilized in the beverage, food, pharmaceutical, perfume, dyestuff, and soap industries. The most important use of benzaldehyde is the manufacturing of different organic products. Benzaldehyde may be released to the ecosystem with emissions of combustion processes such as incinerators, gasoline and diesel engines, and burning of wood. Photochemical oxidation of aromatic hydrocarbons like toluene can be enhanced in the formation of benzaldehyde in the atmosphere. Benzaldehyde can occur naturally in different plants and light has influenced its composition; so, benzoic acid can be produced from oxidizing benzaldehyde in air. Thus, the destruction or removal of benzaldehyde, benzoic acid and other organic compounds related to benzaldehyde from waste streams becomes a major environmental problem [1,2,3].

Many techniques have been developed for the removal of such types of pollutants from wastewater such as advanced oxidation processes [4], aerobic degradation [5], nano-filtration [6], ozonation [7] and others. The familiar technique is the adsorption process which has become one of the most effective means for transferring the solute from the liquid phase to the solid phase of a sorbent [8,9,10,11,12,13]. Adsorption can be achieved by using different material especially the activated carbon which is a very useful and effective technique in the remediation of wastewater contaminated with organic compounds [14,15,16,17]. In this regard, the previous literature was directed towards the adsorption of benzaldehyde using traditional sorbents such as activated carbon cloth [18], granular activated carbon [3], natural clay and Faujasite-Y type zeolite [19]. However, the high cost of the activated carbon and other conventional sorbents were the main constraint that limited their usage; consequently, finding non-conventional low-cost materials with good ability in the reduction of the contamination opens new horizons for many studies. In this direction, byproducts resulted from different industries such as fly ash, bottom ash, and iron slag can be tested to find their suitability for the removal of organic compounds described previously.

The granular iron slag is considered a byproduct solid wastes produced from recycled remnants of iron and steel factories. The previous studies proved that the world production of iron slag is almost 50 × 10^6^ tons per year [20,21]. These large quantities can be accumulated on large areas of land and render it unfit for agriculture purposes. The main constituent of iron slag waste is ash from coke used as a reducing material, silica and other non-ferrous components of iron ore, and limestone auxiliary material. Accordingly, reusing this solid waste in the remediation of wastewater is considered a real application of the sustainability principles. Hence, the primary aim of this study is finding the ability of iron slag byproduct for reclamation of wastewater contaminated with benzaldehyde and specifying the predominant mechanisms of the uptake process.

## 2. Materials and Methods

### 2.1. Sorbent and Contaminant

Granular iron slag byproduct was chosen as reactive material and collected from a steel and iron factory in Talee’a city in Babil Governorate, Iraq. To remove the fine powder, distilled water was used to wash off the iron slag; then, it was dried at 383 K for 24 hrs. Thereafter, it was crushed and particle sizes ranging from 0.6 to 1 mm could be selected for achieving the experiments. The slag was kept in desiccators; however, the following tests were conducted according to the standard methods [13] for characterizing its properties and composition; (i) physical tests (porosity, surface area, and bulk density, and (ii) chemical tests (pH, ash content, X-ray diffraction (XRD), Fourier transform infrared (FT-IR) spectroscopy, scanning electron microscopy (SEM), energy-dispersive spectroscopy (EDS), and X-ray fluorescence (XRF) spectroscopy).

Benzaldehyde (C_7_H_6_O) compound was selected to represent the organic contaminant which has the purity greater than 99%. To prepare the aqueous solution contaminated with certain concentrations of benzaldehyde at room temperature, this compound was dissolved firstly in ethanol (C₂H₆O) and the mixture added to distilled water. The concentration was measured by using gas chromatograph (GC, 1000, Italia). The 0.1 M of sodium hydroxide or nitric acid was used to reach the required value of the pH for the aqueous solution and measurement of this parameter was achieved by pH-meter (WTW, Bench model, German).

### 2.2. Batch Experiments

Certain volumes of C_7_H_6_O aqueous solution (100 mL) with initial concentration (*C_o_*) of 300 mg/L were distributed on the series of 250 mL conical-flasks and 0.3 g of granular iron slag added to each flask. Then, the flasks have been mounted on the high-speed orbital shaker type (Edmund Buhler SM25, German) which operated at shaking speed equal to 250 rpm for 4 h. From each flask, 20 mL of the solution was filtered using a micro filter (Sartorius Stedim 0.2 µm) to separate the solid particles. The residual benzaldehyde (*C_e_*) was measured by gas chromatograph (GC). The batch experiments were conducted with different operational conditions; specifically, initial pH of (3, 4, 5, 6 and 7), initial concentration of (300, 400, 500, 600 and 700 mg/L), sorbent dosage of (0.1, 0.3, 0.5, 0.7, 1.0 g/100 mL) and agitation speed of (0, 50, 100, 150, 200 and 250 rpm). Also, the effects of temperature variation on the sorption performance were investigated at 20, 30 and 40 °C. The quantity of benzaldehyde sorbed onto the sorbent (*q_e_*) was calculated by the following Equation [22]:

(1) $$q_{e}=\left(C_{o}-C_{e}\right)\frac{V}{m}$$

where *V* is the aqueous solution volume added to the flask (L), and *m* is the quantity of granular iron slag in the flask (g). The efficiency of adsorption (*E_ad_*) was calculated in each experiment by:

(2) $$E_{ad}=\frac{\left(C_{o}-C_{e}\right)}{C_{o}}\times 100$$

### 2.3. Kinetic Studies

The kinetic data measured in the batch experimental tests were analyzed by a set of kinetic models mentioned in items from 1 to 3 [23,24]:

Pseudo-first-order model: this is utilized for description the adsorption of solid/solution system with the following exponential form:

(3) $$q_{t}=q_{e}\left[1-exp\left(-k_{1}t\right)\right]$$

where *k*_1_ is the rate constant of the first model (1/min), *q_e_* and *q_t_* are the quantity of contaminant sorbed per unit mass of sorbent (mg/g) at equilibrium and time t respectively.

Pseudo-second order model: it is assumed that (i) the single layer of solute must be sorbed on the solid particle, (ii) the energy of adsorption is not depended on the surface coverage, and (iii) sorbed species must not interact. The expression of the second model has the following non-linear formula:

(4) $$q_{t}=\frac{k_{2}q_{e}^{2}t}{\left(1+k_{2}q_{e}t\right)}$$

where *k*_2_ is the rate constant of second model (g/mg min).

Intra-particle diffusion model: It is depended on the Weber and Morris theory; however, the general formula of the diffusion model relates between qt and *t*^0.5^ is as follows:

(5) $$q_{t}=k_{int}t^{0.5}+C$$

where C is the intercept value and gives an idea about the thickness of the boundary layer, and *k_int_* is the constant for description of adsorption rate in this model (mg/g min^0.5^). The intra-particle diffusion occurred if the plot of Equation (5) takes the linear form; however, this mechanism will be the rate-limiting process when the intercept of the linear plot is equal to zero. Otherwise, additional mechanisms may have occurred with process of intra-particle diffusion.

Frequently, it is recognized that the linear relation of diffusion model can be composed of (i) sharper plot in the first region which reflects instantaneous (or external surface) sorption, (ii) gradually varied plot in the second region and this means that the rate limiting may be the intra-particle diffusion, (iii) the equilibrium plot is in the third region and appeared for cases when this diffusion initiates to slow down because of the extreme decrease in the contaminant concentration within the aqueous solution [25].

### 2.4. Adsorption Isotherm Models

Adsorption model is related between the mass of contaminant per unit mass of sorbent (*q_e_*) and the equilibrium concentration of the contaminant (*C_e_*) [26,27]. A survey for the isotherms applied in this work can be introduced below:

Freundlich model: this is the first known expression for description of sorption data and it has the following formula:

(6) qe=KFCe1n

where 1/*n* (<1) is the sorption intensity, and *K_F_* is reflected the maximum adsorption capacity of the sorbent [22,28].

Langmuir model (1918): this is valid for adsorption of single-layer; however, this model was derived based on the number of assumptions; specifically, the saturation state of all binding sites with molecules of contaminant is equivalent to the maximum adsorption capacity (*q_max_*), the energy of adsorption must be constant, and no transmigration of contaminant within the plane of the solid surface. Langmuir suggested the formula [22,29] below:

(7) $$q_{e}=\frac{q_{max}bC_{e}}{1+bC_{e}}$$

where *b* is the slope of Langmuir relationship and; physically, it means the affinity between the contaminant and the sorbent.

## 3. Results

### 3.1. Description of Iron Slag

**Figure 1:**
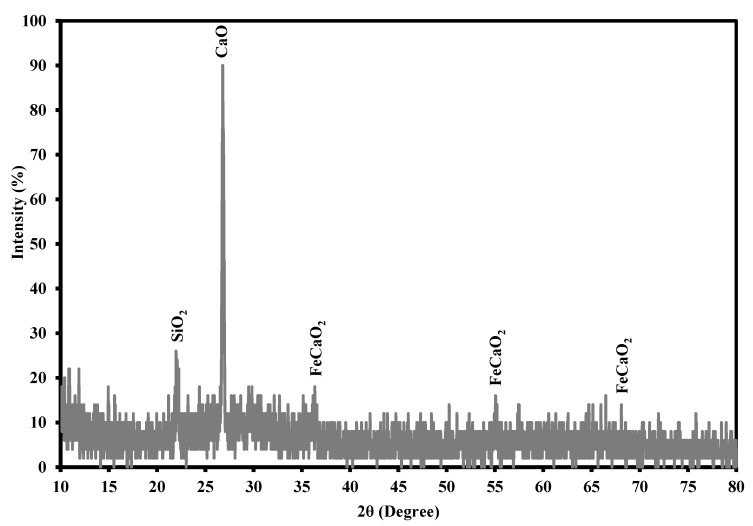
X-ray diffractogram of granular iron slag.

The porosity, bulk density, ash content, pH and Brunauer–Emmett–Teller (BET) surface area of the iron slag has the values of 0.41, 2.026 g/cm^3^, 10%, 8 and 0.2571 m^2^/g respectively. Mineralogical composition of granular iron slag was performed at room temperature using XRD analysis as plotted in Figure 1. This analysis certified that the main components of iron slag are diopside (81.6%), tridymite (9.5%), and silicon oxide quartz (8.9%). Also, XRF analysis was carried out by X-Ray Fluorescence Spectro-Germany to specify the chemical composition of this slag. The outputs of this analysis showed that there is silicon oxide (SiO_2_), iron oxide (Fe_2_O_3_), calcium oxide (CaO), aluminum oxide (Al_2_O_3_) and manganese oxide (MnO) with percentages reached to 31.2, 9.95, 13.48, 7.933, and 6.853%, respectively, in the composition of iron slag.

### 3.2. Operational Conditions

#### 3.2.1. Equilibrium Time and Initial pH of the Solution

**Figure 2:**
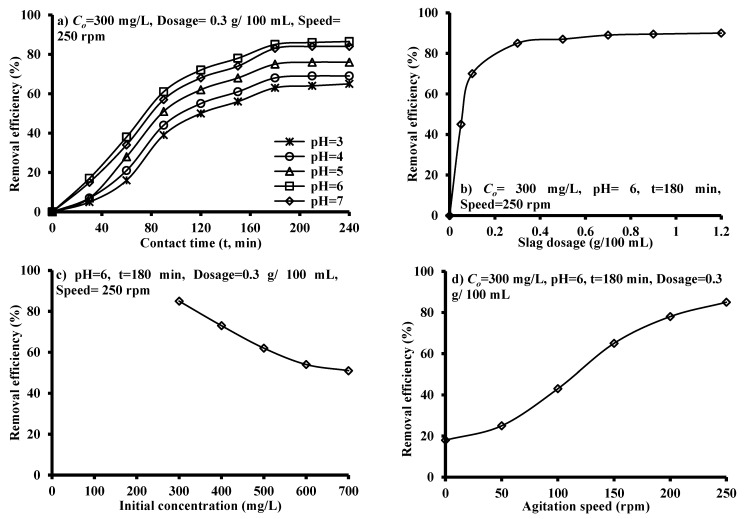
Effect of (**a**) contact time and initial pH, (**b**) iron slag dosage, (**c**) initial concentration and (**d**) agitation speed on the removal efficiencies of benzaldehyde from contaminated solution.

The behavior of removal efficiency of the benzaldehyde under the effect of contact time was studied by adding 0.3 g of granular iron slag to 100 mL of contaminated solution at room temperature for initial pH changes from 3 to 7 and measured values are plotted in Figure 2a. In spite of the difference of the initial pH, all curves show the same trend. At initial time until 180 min, rapid increase in the sorption rate can be observed; thereafter, the rate is gradually slowed and this behavior may be due to decrease of the binding sites. The kinetic data measured for interaction of aqueous solution contaminated with benzaldehyde and iron slag illustrated that the best equilibrium is equal to 180 min with maximum removal efficiency of 85%. However, no change can be recognized in the residual concentration beyond this time and this means that all remaining batch experiments can be conducted with time equal to 180 min.

Also, Figure 2a proved that the removal efficiency is influenced significantly by the values of initial pH where it increased from 63% at pH of 3 to become 85% at pH of 6, and then decreased to 83% at pH of 7 at equilibrium time. This trend of variation is applicable for all measurements as a result of initial pH variation at certain time. The chemical speciation of organic compounds is affected by pH of water which caused a change in their adsorption properties on granular iron slag. The variations of pH would affect the zeta potential [30] and forms of functional groups [31,32] responsible for benzaldehyde removal onto the iron slag. The balance between repulsive and attractive forces can be affected by pH of solution which has significant influence on the adsorption of organic compounds. The protonation–deprotonation transition of these compounds will increase the affinity of adsorption until pH equal to 6 because this value will enhance the electrostatic adsorption. It is important to know whether there will be any leaching from the iron slag when the slag utilized for treating of water. The two leaching tests are implemented with dosage of 0.3 g/100 mL, speed of 250 rpm and contact time of 180 min for two values of pH 3 and 6 to find the heavy metals concentrations in the leachate. The results of these tests (Table 1) proved that there are no significant concentrations of heavy metals in the resulting leachate.

#### 3.2.2. Slag Dosage and Initial Concentration

Figure 2b reveals that the increase of iron slag dosage will have positive effect on the sorption of benzaldehyde where variation of the dosage from 0.05 to 0.3 g for 100 mL of aqueous solution will be associated with increase in uptake efficiency from 45% to 85% respectively. This behavior is expected because the higher dosage of sorbent means the higher binding sites; however, the increase of sorbent dosage beyond 0.3 g/100 mL will not have significant effect on the residual concentration because this concentration reaches a low value. In addition, the relationship between the *C_o_* of benzaldehyde and the efficacy of the sorption process was investigated within the range (300–700 mg/L) as plotted in Figure 2c. This plot illustrates that the increase of initial concentration will cause a clear reduction in the sorption efficiency because of the stabilization of the binding sites number [26].

#### 3.2.3. Agitation Speed

The sorption efficiency of benzaldehyde onto iron slag due to variation of shaking speed was investigated within the range from zero to 250 rpm with *C_o_* = 300 mg/L, pH = 6, *t* = 180 min, dosage = 0.3 g/100 mL. The relationship is plotted in Figure 2d which proves that the removal increased from 18% to 85% when the speed changed from zero to 250 rpm respectively. This is logical behavior for a relation between removal efficiency and agitation speed because the agitation will achieve proper contact between the contaminant in the liquid phase and solid particles; therefore, this will increase the contaminant diffusion towards the surface of these particles.

### 3.3. Models of Kinetic Studies

**Figure 3:**
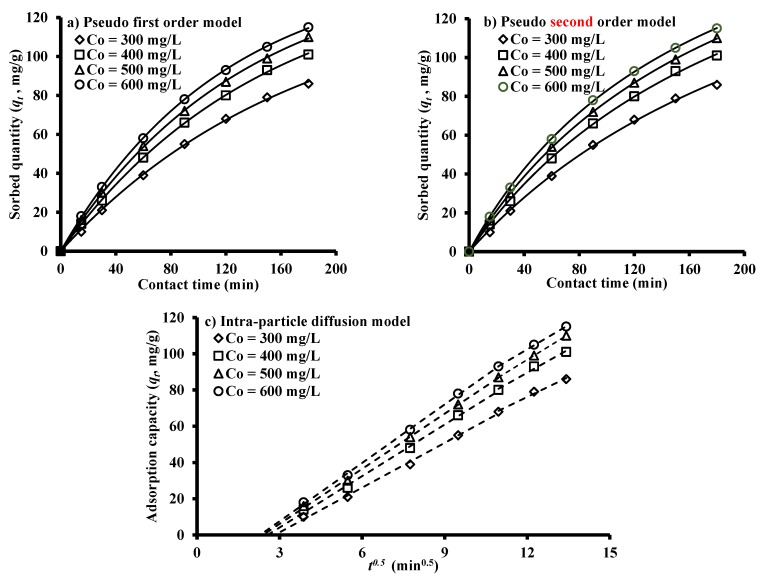
Kinetic models for sorption of benzaldehyde onto iron slag sorbent.

Kinetic studies were employed to identify the mechanism and sorption rate for removal of benzaldehyde from aqueous solution onto iron slag. These experiments were carried out at sorbent dosage of 0.3 g/100 mL, initial pH of 6, and agitation speed of 250 rpm for initial concentrations of 300, 400, 500 and 600 mg/L and contact time not exceeding 180 min. Pseudo-first and -second order models are fitted with these data (Figure 3a,b) to interpret the mechanisms responsible for the sorption process [33,34]. When the pseudo-first-order is applicable to the kinetic data, this means that there is linear variation for the rate constant with contaminant concentration [35,36]. However, the pore diffusion will govern the adsorption process in the case where the relationship is non-linear [37]. Origin 2018 software was applied to find the constants of the applied models (Table 2) for system of benzaldehyde and iron slag using non-linear regression analysis. Figure 3a,b with Table 2 signified that the interaction of benzaldehyde and iron slag can be well described by the pseudo-first order model because the adsorption capacity approaches from the capacity specified by the equilibrium sorption. Hence, the physical forces are predominant in the sorption of benzaldehyde onto iron slag; also, there is obvious concurrence between the predicted and experimental values.

To specify the role of film diffusion and intra-particle diffusion in the removal of benzaldehyde onto iron slag, the model of intra-particle diffusion plots is shown in Figure 3c. This graph shows that the plot is consisted of two regions and intercept with *y*-axis cannot be equal to zero. Consequently, the rate limiting step must not govern by intra-particle diffusion only and there is more than one way to describe the sorption mechanism. In the initial stages of removal process, the external mass transfer was predominant [38]; however, the presence of intra-particle diffusion results in the gradual decrease of adsorption in the second linear portion [39]. In addition, there are sufficient vacant sites at the beginning of the sorption process to increase the removal rate and this can be recognized through higher values of slopes for 1st portion (Table 2). Conversely, the lowest slopes (Table 2) for the 2nd portion means that the removal rate or diffusion of contaminant into the micro-pores is slow and this may be due to decrease of the concentration gradient.

### 3.4. Sorption Isotherms

**Figure 4:**
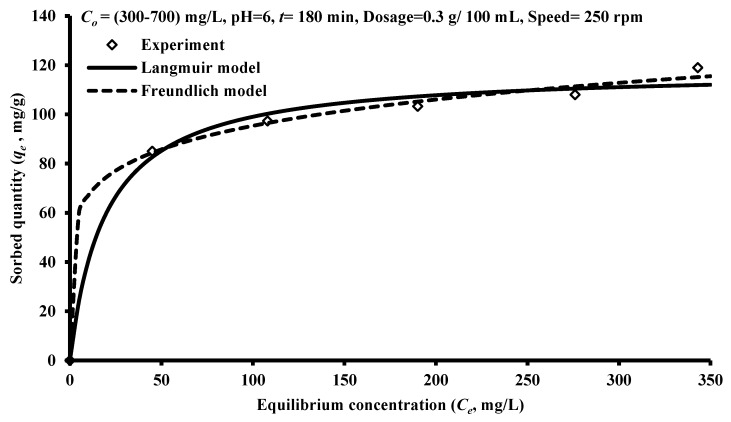
Comparison of the experimental results with the *q_e_* values predicted by Freundlich and Langmuir isotherm models for benzaldehyde removal by iron slag.

The Origin 2018 software was utilized for calculating the constants of Freundlich and Langmuir models through fitting them with sorption measurements as plotted in Figure 4 using non-linear regression analysis. Outputs of the fitting process (i.e., constants of isotherms and determination coefficients (*R*^2^)) are inserted in Table 3; however, the two models used are able to describe the interaction of benzaldehyde and iron slag well with *R*^2^ > 0.86. Based on Langmuir model, the maximum sorbed quantity of benzaldehyde per unit mass of iron slag was reached to 118.25 mg/g.

### 3.5. Thermodynamics of Adsorption Process

For the sorption process under consideration, the parameters of thermodynamic (specifically Gibbs free energy change ($∆G^{{}^{\circ}}$), entropy change ($∆S^{{}^{\circ}}$), and enthalpy change ($∆H^{{}^{\circ}}$)) have been calculated based on the variation of Langmuir constant (b) with temperature by applying the following relationships:

(8) $$∆G^{{}^{\circ}}=RTln\left(b\right)$$

(9) $$∆G^{{}^{\circ}}=∆H^{{}^{\circ}}-T∆S^{{}^{\circ}}$$

(10) $$ln\left(k_{c}\right)=\frac{∆S^{{}^{\circ}}}{R}-\frac{∆H^{{}^{\circ}}}{RT}$$

where *T* is the absolute temperature (K), (*k_c_*, L/g) is the coefficient of thermodynamic distribution for the adsorption which is calculated by the division of *q_e_* into *C_e_* and *R* is the universal gas constant (8.314 × 10^−3^ kJ mol^−1^ K^−1^).

**Figure 5:**
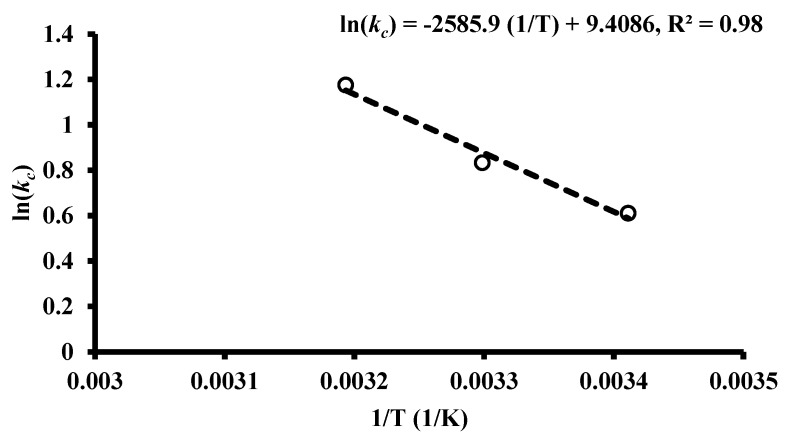
Liner plot related between ln(*k_c_*) and 1/T for adsorption of benzaldehyde onto iron slag.

The linear relationship between ln$k_{c}$ and 1/T can be used to evaluate the enthalpy change for sorption of benzaldehyde into iron slag for adopted range of temperature by applying the least square analysis as shown in Figure 5. For different values of temperature, Table 4 presents the calculated magnitudes of $∆G^{{}^{\circ}}$, $∆H^{{}^{\circ}}$, and $∆S^{{}^{\circ}}$ for benzaldehyde adsorption onto iron slag. The positive $∆H^{{}^{\circ}}$. suggests an endothermic adsorption process; however, with temperature increase $∆S^{{}^{\circ}}$. becomes more negative. This reveals that the sorption process is spontaneous and may be enhanced with higher temperature. Moreover, the $∆S^{{}^{\circ}}$ with positive value reveals that there is random increase in the sorbed contaminant at the solid/liquid interface with high affinity.

## 4. Predominant Mechanisms

**Figure 6:**
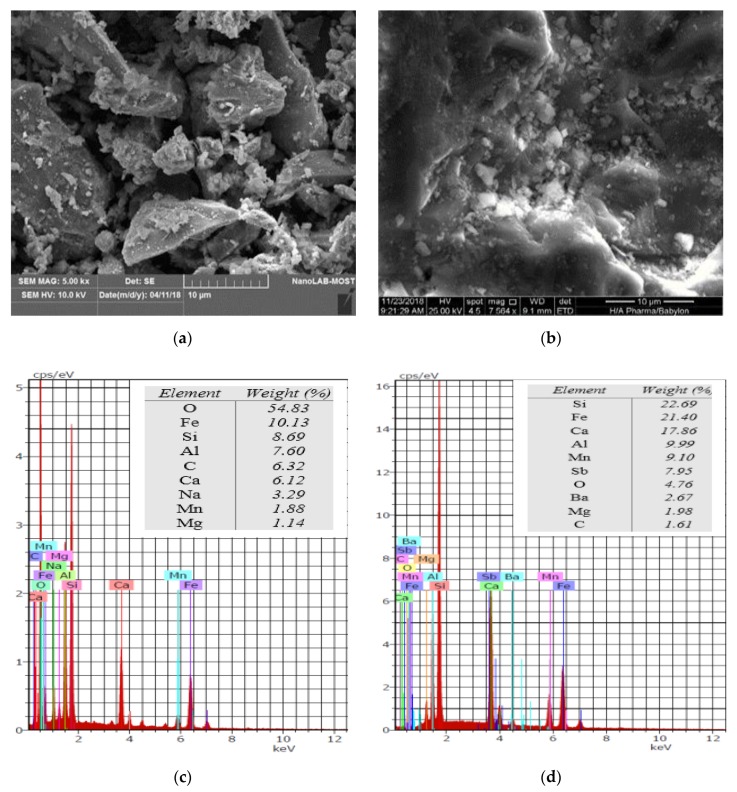
Scanning electron microscopy/energy-dispersive spectroscopy (SEM/EDS) for iron slag (**a**), (**c**) before and (**b**), (**d**) after adsorption of benzaldehyde from aqueous solutions.

The most important property of the adsorbent is the surface morphology and the difference in the porosity and structure of this surface can be considered the base for generation of the adsorption capacity. SEM/EDS tests were conducted to investigate the shape and surface morphology of iron slag before and after adsorption of benzaldehyde and results are shown in Figure 6. This figure confirms that the surfaces are irregular and this will enhance the adsorption capacity; however, the higher surface area may have resulted from the dissolution of calcium oxide. These results are proved through measurement of the final pH and concentration of calcium after adsorption process in comparison with those values in the control flask (i.e., sorbent plus deionized water) as illustrated in Table 5. This table reveals the increase of calcium concentration when the initial pH changed from 3.17 to 6.25; however, this concentration was decreased in the basic range of pH. Based on the output of EDS analysis in Figure 6, significant change in the chemical composition of the iron slag can be observed due to the formation of bridge cations in the presence of large calcium concentration and this may be the cause of the difference in final pH between and after adsorption and control tests.

**Figure 7:**
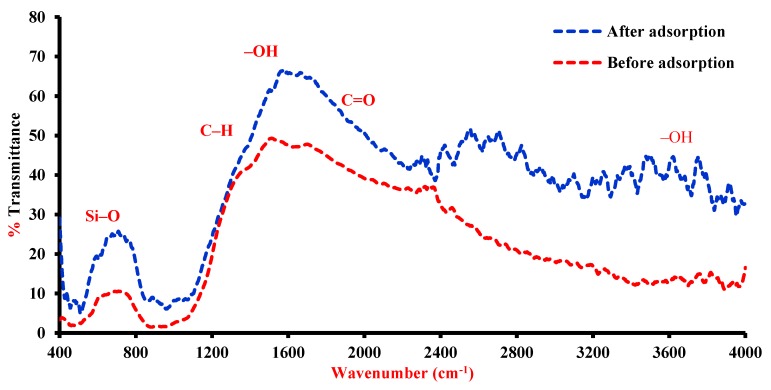
Fourier transform infrared (FT-IR) spectra for iron slag before and after adsorption process.

The infrared spectrum of slag before and after adsorption is plotted in Figure 7 and it is clear that the absorption bands of Si–O, Al–O, Mg-O, Fe-O and Ca–O vibrations appear at wavelengths of 3622, 1635, 1421, 996, 872, 727, 530, and 450 cm^−1^ (Table 6) [40]. Stretching bands near 1421 and 872 cm^−1^ represent the existence of calcium with carbonates species and this related with the presence of calcite; however, this is consistent with XRF results. At a wavelength of 3622 cm^−1^, the surface hydroxyl groups (Si–Si–OH, or Al–Al–OH coupled by A1MgOH) can be recognized, while the bending of sorbed water between the layers can be generated at 1635 cm^−1^. Vibrations of SiO_4_ could correspond with bands at 996, 530 and 450 cm^−1^. Maximum absorption of silicate minerals was observed at 996 cm^−1^ while bands at 450 and 530 cm^−1^ could be Si–O–Si and Al–O–Si bending vibrations, respectively. The structure of the molecules under consideration consists of the benzene ring to which the C=O group and the hydrogen atom are bonded; in addition, various substituents can be bonded to different positions of the ring. As a rule, to identify compounds that contain the aldehyde–CHO group, their infrared (IR) spectra are studied in detail in the range 1760–1640 cm^−1^, in which strong absorption bands of C=O vibrations are observed, and in the range 2900–2700 cm^−1^, where characteristic absorption bands of stretching vibrations of the aldehyde C–H group are located [41,42,43]. Figure 7 signifies that the spectrum of aqueous solution with benzaldehyde includes a very strong band at 1623 cm^−1^ identical to the C=O bond [43]. For adsorbed benzaldehyde, this band is also available in the spectra and this suggests that it belongs to a weakly adsorbed/physisorbed species. During the adsorption of benzaldehyde, new shoulder observes at 1625 cm^−1^ due to vibration of a weakened C=O bond in a covalent carboxylate-type structure [44,45]. For aqueous solution with benzaldehyde after adsorption, the disappearance of a band at 1383 cm^−1^ is supported by the breakage of the aldehydic C–H bond. This band has been previously assigned to a bending vibration of the aldehydic C–H bond [46,47]. Through the sorption of substituted benzaldehyde onto iron oxides, Kung and McBride [48] proved that there are two bands in this region and they identified them as the symmetric stretching vibration of different benzoate species. The authors proposed that these bands may or may not be resolved based on the substituent groups present and they identical to the two benzoate species at wavelengths of 1560 and 1495 cm^−1^. Also, Vanhengstum [44] identified two bands for surface benzoate species coordinated to a single surface metal ion at 1500 and 1410 cm^−1^. At 1493 cm^−1^, a weak band may be assigned to the asymmetric stretching vibration of the same species.

However, the properties in this range (Ca 1470–1530 cm^−1^) cannot be distinguished easily because of the strong IR beam absorbance by the sample. Bands at 1160, 1175, 1306, 1447, and 1601 cm^−1^ are shifted slightly after adsorption in comparison with their original positions. This may result from the vibrations of pure aromatic ring as well as to mixed modes of CH in plane bending and vibrations of C=C stretching [44,49,50]. This means that this ring stays intact through the interaction of slag sorbent and benzaldehyde.

## 5. Conclusions

Due to the use of solid waste (i.e., iron slag) in the reclamation of wastewater, the present study is considered a real application for the sustainability principles. The outputs of batch studies confirmed that this material is more effective and dependable for removing of benzaldehyde present in water with removal efficiency and maximum adsorption capacity reached to 85% and 118.25 mg/g respectively. These values are achieved at best operational conditions of; slag mass of 0.3 g added to 100 mL of contaminated water, initial pH of 6, time of 180 min, shaking speed of 250 rpm for initial concentration of 300 mg/L. The isotherm models in conjugation with kinetic studies elucidated that the physical sorption has a major role in the removal process. However, thermodynamic analysis certified that the sorption process is spontaneous and endothermic. All characterization tests (i.e., SEM, EDS, and FT-IR) elucidated that there is a significant dissolution in the calcium oxide (which formed 13.48% of natural slag) and this will increase the surface area. The increase of dissolved calcium concentration may be responsible for bridge cation formation to eliminate the contaminant for aqueous solution. Also, the outputs of FT-IR analysis signified that the aromatic ring stays intact through the interaction of iron slag and benzaldehyde.

## Figures and Tables

**Table 1:** Leachate from iron slag using deionized water.

Element	Concentration (mg/L)	
	pH 3	pH 6
Pb	0.01	0.008
Cr	n.d.	n.d.
Cu	0.072	0.02
Zn	n.d.	n.d.
n.d.: not detected		

**Table 2:** Kinetic parameters for the adsorption of benzaldehyde onto iron slag.

Kinetic Model	Parameter	*C_o_* (mg/L)			
		300	400	500	600
Pseudo First-Order	*k*_1_ (min^−1^)	0.0058	0.0067	0.0075	0.0084
	*q_e_* (mg/g)	134.5	144.6	147.6	147.0
	*R* ^2^	0.999	0.999	0.999	0.999
Pseudo Second-Order	*k*_2_ (g/mg min)	0.000016	0.00002	0.000022	0.000026
	*q_e_* (mg/g)	225.0	225.9	230.3	224.5
	*h* (mg/g min)	0.796	1.029	1.165	1.314
	*R* ^2^	0.999	0.999	0.999	0.999
Intra-Particle Diffusion	First Portion				
	*k_int_* (mg/g min^0.5^)	8.243	9.466	10.131	10.74
	*R* ^2^	0.997	0.998	0.999	0.999
	Second Portion				
	*k_int_* (mg/g min^0.5^)	7.332	8.557	9.341	8.942
	*R* ^2^	0.990	0.989	1.00	0.999

**Table 3:** Constants of isotherm models with statistical measure for sorption of benzaldehyde onto iron slag.

Isotherm Model	Parameter	Value
Freundlich	*K_F_* (mg/g)(L/mg)^1/*n*^	47.12
	1/*n*	0.153
	*R* ^2^	0.952
Langmuir	*b* (L/mg)	0.052
	*q_max_* (mg/g)	118.25
	*R* ^2^	0.865

**Table 4:** Values of thermodynamic parameters for adsorption of benzaldehyde onto iron slag.

Thermodynamic Parameters	Temperature (K)		
	293.15	303.15	313.15
$k_{c}$	1.84058	2.29825	3.2381
Δ*G*° (* 10^3^ cal mol^−1^)	−0.3554	−0.5013	−0.7311
Δ*H*° (* 10^3^ cal mol^−1^)	5.81494		
Δ*S*° (cal mol^−1^ K^−1^)	18.69577		

**Table 5:** The values of final pH and residual concentration of calcium after the sorption of benzaldehyde onto iron slag in comparison with the control test.

Initial pH	Control Test		After Adsorption	
	Final pH	Ca^+2^ (mg/L)	Final pH	Ca^+2^ (mg/L)
3.17	4.39	3.4	3.76	1.3
5.9	6.49	3.2	6.3	2
6.25	8.20	10	6.75	4
6.98	8.5	7	7.24	5
8.87	6.5	2.6	7.4	0.5
11	10.7	0.5	10.6	0.54

**Table 6:** Functional groups responsible of benzaldehyde onto iron slag.

Band (cm^−1^)	Functional Group
996,530 and 450	Si–O
450	Ca–O
1421 and 872	C-O
3622	Si–Si–OH or Al–Al–OH
1635	A1MgOH
